# Corrigendum to: Expression of the chloroplast thioredoxins *f* and *m* is linked to short-term changes in the sugar and thiol status in leaves of *Pisum sativum*

**DOI:** 10.1093/jxb/erz338

**Published:** 2019-09-11

**Authors:** Juan De Dios Barajas-López, Justyna Tezycka, Claudia N Travaglia, Antonio Jesús Serrato, Ana Chueca, Ina Thormählen, Peter Geigenberger, Mariam Sahrawy

**Affiliations:** 1 Departamento de Bioquímica, Biología Molecular y Celular de Plantas, Estación Experimental del Zaidín, Consejo Superior de Investigaciones Científicas, Granada, Spain; 2 Ludwig-Maximilians-Universität München, Department Biology I, Martinsried, Germany; 3 Departamento de Ciencias Naturales, Facultad de Ciencias Exactas, Físico Químicas y Naturales, Universidad Nacional de Río Cuarto, Campus Universitario, Río Cuarto, Argentina

*Journal of Experimental Botany,* (2012) 63(13): 4887-4900, doi: 10.1093/jxb/ers163

In the above article several bands of the western blottings of figures 4 and 5 (anti TRXs *f* and anti TRXs *m*) were inadvertently duplicated. The experiment was repeated and figures 4 and 5 have been substituted.

The figures now appear as below:

**Figure 4.**
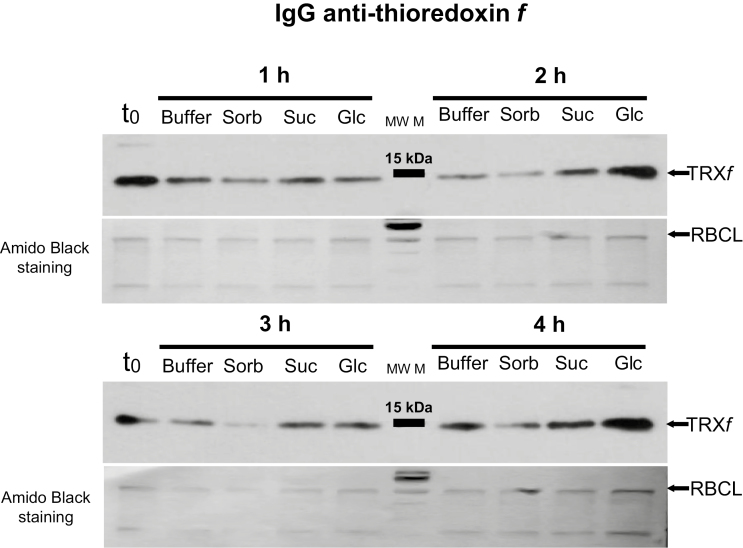
Short-term feeding of sugars to leaf discs of pea plants at the end of the night leads to increased TRX *f* protein level. Leaves of pea plants were harvested at the end of the night to prepare discs that were immediately incubated in the dark for 1, 2, 3 and 4 h in buffer (control), 100 mM sorbitol (Sorb), 100 mM sucrose (Suc) or 100 mM glucose (Glc), before samples were frozen to analyse TRX *f* protein level using Western blots. t0: leaf tissue before incubation. Western blot results images are representative of two independent experiments.

**Figure 5.**
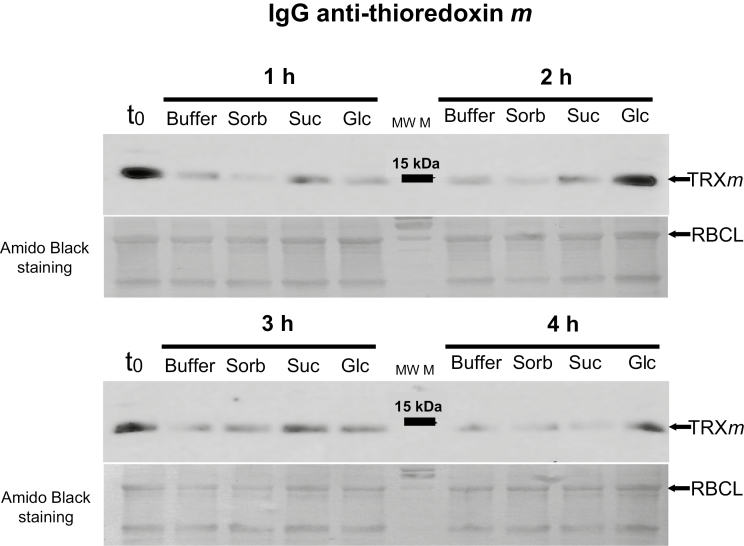
Short-term feeding of sugars to leaf discs of pea plants at the end of the night leads to increased TRX *m* protein level. Leaves of pea plants were harvested at the end of the night to prepare discs that were immediately incubated in the dark for 1, 2, 3 and 4 h in buffer (control), 100 mM sorbitol (Sorb), 100 mM sucrose (Suc) or 100 mM glucose (Glc), before samples were frozen to analyse TRX *m* protein level using Western blots. t0= leaf tissue before incubation. Western blot results images are representative of two independent experiments.

The authors apologize for these errors which have been corrected online.

